# Phlorofucofuroeckol Improves Glutamate-Induced Neurotoxicity through Modulation of Oxidative Stress-Mediated Mitochondrial Dysfunction in PC12 Cells

**DOI:** 10.1371/journal.pone.0163433

**Published:** 2016-09-26

**Authors:** Jwa-Jin Kim, Yoon-Joong Kang, Sun-Ae Shin, Dong-Ho Bak, Jae Won Lee, Kyung Bok Lee, Yung Choon Yoo, Do-Kyung Kim, Bong Ho Lee, Dong Woon Kim, Jina Lee, Eun-Kyeong Jo, Jae-Min Yuk

**Affiliations:** 1 Department of Biomedical Science, Jungwon University, Geosan, Chungbuk, South Korea; 2 Material Science and Engineering, Jungwon University, Geosan, Chungbuk, South Korea; 3 Department of Anatomy, College of Medicine, Konyang University, Daejeon, South Korea; 4 Biochemistry, College of Medicine, Konyang University, Daejeon, South Korea; 5 Microbiology, College of Medicine, Konyang University, Daejeon, South Korea; 6 Pharmacology, College of Medicine, Konyang University, Daejeon, South Korea; 7 Department of Chemical and Biological Engineering, Hanbat National University, Daejeon, South Korea; 8 Department of Medical Science, College of Medicine, Chungnam National University, Daejeon, South Korea; 9 Microbiology, College of Medicine, Chungnam National University, Daejeon, South Korea; 10 Infection Biology, College of Medicine, Chungnam National University, Daejeon, South Korea; 11 Anatomy, College of Medicine, Chungnam National University, Daejeon, South Korea; 12 Brain Research Institute, College of Medicine, Chungnam National University, Daejeon, South Korea; 13 LES Corporation Inc., Daejeon, South Korea; University of PECS Medical School, HUNGARY

## Abstract

Stroke is a complex neurodegenerative disorder with a clinically high prevalence and mortality. Despite many efforts to protect against ischemic stroke, its incidence and related permanent disabilities continue to increase. In this study, we found that pretreatment with phlorofucofuroeckol (PFF), isolated from brown algae species, significantly increased cell viability in glutamate-stimulated PC12 cells. Additionally, glutamate-stimulated cells showed irregular morphology, but PFF pretreatment resulted in improved cell morphology, which resembled that in cells cultured under normal conditions. We further showed that PFF pretreatment effectively inhibited glutamate-induced apoptotic cell death in a caspase-dependent manner. Reactive oxygen species (ROS) induced by oxidative stress are closely associated with ischemia-induced neurological diseases. Exposure of PC12 cells to glutamate induced abundant production of intracellular ROS and mitochondrial dysfunction, which was attenuated by PFF in a dose-dependent manner. *In vivo* studies revealed that PFF-mediated prevention was achieved predominantly through inhibition of apoptosis and mitochondrial ROS generation. Taken together, these results suggest the possibility of PFF as a neuroprotective agent in ischemic stroke.

## Introduction

Ischemic stroke, the most common type of stroke, is among the leading causes of long-term disability and mortality [1, 2]. The dramatic disruption of the bloodstream that occurs for minutes in ischemic stroke leads to deficiencies in essential nutrients because of thrombosis or embolism [3]. According to World Health Organization (WHO) statistics, the global stroke burden has increased significantly over the last 20 years, and about 15 million people suffer a stroke each year [4]. There have been many efforts to cure stroke; however, there is still no safe and effective therapy for this condition. Although thrombolytic are the only treatment approved by the Food and Drug Administration (FDA), they are useful in only limited situations due to the short time requirement for administration and the high risks for later treatment [5]. Thus, the discovery and development of novel neuroprotective drugs for ischemic stroke is important.

Oxidative stress is a well-known common hallmark closely implicated in the progression of lifestyle-related diseases, including obesity, ischemic disease, atherosclerosis, and arthritis [6]. There is emerging evidence that reactive oxygen species (ROS) are generated in various mammalian cells upon interaction with environmental stress and play roles as signaling molecules in neuronal cells [7–9]. Accumulation of ROS has been associated with mitochondrial dysfunction, impaired maintenance of energy metabolism and metal homeostasis, and increased protein aggregation in neurodegenerative disorders [8, 10, 11]. Of the many neurodegenerative pathways, oxidative stress-mediated neuronal cell death is one of the main processes exacerbating ischemic stroke, which is mediated by an imbalance between antioxidant systems and the production of free radicals [12–14]. Thus, the development of novel antioxidants for regulating oxidative stress and redox imbalance may be an appropriate approach to combating ischemic stroke.

*Ecklonia cava*, an edible marine brown alga, is commonly distributed on the southern coast of Korea and contains various bioactive derivatives, including phlorotannins, sulfated polysaccharides, peptides, carotenoids, and fucoidans [15]. Previous studies have shown that phlorotannins, marine brown algal polyphenols, possess valuable biological features involved in regulating the generation of ROS [16–18], inflammation [19], and host immune responses against bacterial [20] and human immunodeficiency virus type-1 (HIV-1) [21] infection. Additionally, phlorofucofuroeckol (PFF), a type of phlorotannin, can be isolated from *Ecklonia* species and also exhibits multifunctional properties [22, 23]. Recent studies have considered PFF a potential candidate for the treatment of Alzheimer’s disease, as it modulates acetylcholinesterase activity [24] and inhibits intracellular ROS and Ca^2+^ generation [25]. Although these studies provide insight into the possible use of PFF in Alzheimer’s disease, functional studies regarding its specific role in the treatment of ischemic stroke remain at any early stage.

In the study, we sought to characterize the role of PFF isolated from *Ecklonia cava* Kjellman in the regulation of glutamate-induced neurotoxicity in PC12 cells.

## Materials and Methods

### Cell Culture

PC12 cells derived from pheochromocytoma of the rat adrenal medulla were obtained from the Korean Cell line Bank (Seoul, Korea). PC12 cells were seeded at a density of 2×10^6^ / dish in 100mm dishes (Falcon; Becton-Dickinson, Oxnard, CA) in DMEM (Life Technologies-Invitrogen) with 10% heat-inactivated (56°C for 0.5 h) fetal bovine serum (FBS) and antibiotics at 37°C in a humidified atmosphere of 5% CO2. Cells were sub-cultured twice a week, and only those in the exponential growth phase were used in experiments. PC12 cells were subsequently incubated with 1 μM retinoic acid (Sigma Aldrich, USA) in 10% FBS containing DMEM for 24 hours.

### Animal

Animal experimental procedures were approved by the Institutional Animal Care and Use Committee (IACUC) at Chungnam national university (CNUH-014-A0008) and conformed to National Institutes of Health guidelines. The animals were fed standard rodent food and water ad libitum, and housed (maximum of 3 per cage) in sawdust-lined cages in an air-conditioned environment with 12-hour light/dark cycles. Animal husbandry was provided by the staff of the IACUC under the guidance of supervisors who are certified Animal Technologists, and by the staff of the Animal Core Facility. Veterinary care was provided by IACUC faculty members and veterinary residents located on the Chungnam National University School of Medicine.

For surgeries, the animals were anesthetized with 2% isoflurane inhalation, and given acetaminophen with drinking water one day before and one day after surgical procedures. Buprenorphine (0.1 mg/kg body weight) was injected intra-peritoneally to reduce pain: the first dose at the beginning of the procedure, the second dose 4–6 hours later, and then every 8–12 hours as needed. An animal's ability to drink water, feed, ambulate, guard, painful areas, and its general overall appearance were evaluated five minutes after surgery.

Animals were placed in their home cages when they were able to ambulate, feed, and drink unaided. They were monitored twice a day on the first day, daily during the first week, and then 3 times a week until they were euthanized. The monitor criteria included: (1) wound inflammation diagnosed by swelling, edema or dehiscence; (2) wound bleeding diagnosed by local enlargement; (3) pain diagnosed by body movement and gesture; (4) neurological defects, abnormal gait, hemiplegia, or coma; (5) body weight loss; and (6) foot necrosis.

For all cases of wound enlargement, inflammation, bleeding or pain, veterinary staff was consulted to determine whether the animal should be treated or euthanized. The staff’s instructions were followed as to the kind and dose of antibiotics to be used for the particular infection. If the animals were not responsive to treatment, or if there was any evidence of post-operative respiratory compromise or bleeding, they were euthanized. In our experiments, none of the mice became so severely ill that they needed to be removed before the experiments ended.

### The Preparation of PFF

The seaweed (1000 g) was extracted with 95% ethanol (10 L) for 3 h in a water bath at 50°C. The solvent was evaporated in vacuo to give a gummy extract, which was partitioned between ethyl acetate and water. The ethyl acetate soluble portion was subjected to ODS (Octadecylsilane) column chromatography followed by gel filtration on Sephadex LH-20 with methanol. Final purification of individual compound was accomplished by HPLC (Waters Spherisorb S10 ODS2 column (20 × 250mm); eluent, 30% MeOH; flow rate, 3.5mL/min). By means of 1H-NMR spectra and comparison with published data, phlorofurofukoeckol-A (PFF-A, C5) were identified. Phlorofurofukoeckol-A: white solid; UV (EtOH) λmax 227 nm; 1H-NMR (DMSO-d6, 500MHz) δ 5.73 (d. J = 1.85, 1H), 5.76 (d, J = 1.85, 1H), 5.83 (t, J = 1.85, 1H), 5.84 (t, J = 1.85, 1H), 6.29 (s, 1H), 6.43 (s, 1H), 6.72 (s, 1H), 8.22 (s, OH), 9.19 (s, OH), 9.22 (s, OH), 9.45 (s, OH), 9.86 (s, OH), 9.88 (s, OH), 10.15 (s, OH); 13C-NMR (DMSO-d6, 125MHz) δ 94.3, 94.0, 95.3, 96.9, 97.0, 98.8, 99.6, 103.8, 103.9, 120.6, 123.0, 123.1, 126.9, 134.5, 137.4, 142.5, 145.3, 147.0, 147.5, 150.0, 150.9, 151.4, 159.4, 159.5, 160.5, 160.8. FABMS m/z 603.1 (M+H)+.

### Reagents and Antibody

All chemicals and solvents used were of the highest analytical grade available. Cell culture supplies and media including FBS, phosphate-buffered saline (PBS), and penicillin-streptomycin were purchased from Thermo (Waltham, MA, USA). 3-(4,5-dimethylthiazol-2-yl)-2,5-diphenyltetrazolium bromide (MTT) and Hoechst 33258 and protease inhibitor cocktail were purchased from Sigma—Aldrich (St. Louis, MO, USA). Annexin V-FITC was purchased from BD biosciences (Flanklin Lakes, NJ, USA). Dihydroethidium (DHE) was purchased from Calbiochem (Billerica, MA, USA). Tetramethylrhodamine (TMRE), MitoSOX^™^ Red and Mito Tracker Green FM were purchased from Thermo (Waltham, MA, USA). The BCA protein assay kit was purchased from Pierce (Lockford, IL, USA). The enhanced chemiluminescence (ECL) detection kit was acquired from Amersham Pharmacia (Arlington Heights, IL). Pan-actin was obtained from Cell Signaling Technology (Beverly, MA, USA). Anti-cleaved caspase 3 antibody was purchased from EMD Millipore (Darmstadt, Germany). Cy-3 conjugated goat anti-rabbit IgG, anti-mouse, and anti-rabbit IgG horseradish peroxidase (HRP) antibodies were purchased from Santa Cruz Biotechnology, Inc. (Santa Cruz, CA, USA).

### The Morphological Analysis and 3-(4,5-dimethylthiazol-2-yl)-2,5-diphenyltetrazolium Bromide (MTT) Assay

The morphology of PC12 cells was monitored using an inverted contrast phase microscope (Nikon TE 2000-U; Nikon Tokyo, Japan). To determine glutamate half maximal inhibitory concentration (IC_50_ value) on PC12 cell, 5 x 10^3^ cells in single cell suspensions were seeded in individual wells of 96-well plates and incubated for 24 hours at 37°C prior to glutamate treatment at the indicated concentrations (1, 5, 10 mM) for 24 hours. MTT solution was added to each well and incubated for 4 hours at 37°C prior to removing the culture medium. DMSO was then added and mixed for 30 min at room temperature. Cell viability was determined by measuring the absorbance at 562 nm. The cell viability for each group was calculated as a percentage of that of the control group.

### Apoptosis and Cell Death

The protective effects of PFF on glutamate-induced cell damage, were applied to each experimental groups of PC12 cells (Glut, PFF, Glut + PFF) with individual treatments at final concentrations of PFF (10 μM) and glutamate (5 mM) for 24 hours. Dead cells were analyzed using Annexin V-FITC apoptosis detection kit (Enzo, ENZ-51002-100, NY, USA) and the Muse^™^ Annexin V and Dead Cell Assay kit (Muse^™^Cell Analyzer; Millipore, Billerica, MA, USA) according to the manufacturer’s instructions. Briefly, Annexin V-FITC (5 μL) was added into the culture dish and incubated at room temperature in the dark for 10 min. After adding 500 μL of binding buffer, the cells were directly visualized by Confocal microscopy (Zeiss, Germany). The green fluorescence intensity was analyzed from individual group.

Morphological observation of nuclear change was assayed with Hoechst 33258 staining according to the manufacturer’s instructions. In brief, PC12 cells (1×10^4^cells/ml) were seeded in 12-well plates and incubated with individual treatments for 24 hours at 37°C. The cells were collected, washed and fixed in 4% paraformaldehyde for 30 min and then stained with Hoechst 33258 (5 μg/ml) for 5 min at room temperature. The nucleus fragmentations were visualized using a confocal microscopy microscope (Zeiss, Germany).

### Fluorescence Microscopy

Cells (1 × 10^6^ cells/well) were prepared on sterilized glass coverslips (BD Bio-sciences, Bedford, MA, USA) in triplicate and cells then were fixed in 4% paraformaldehyde in PBS for 10 min, permeabilized with 0.25% Triton X-100 in PBS for 10 min, and incubated with primary antibody (Tomm20, ab56783, Abcam, Cambridge, MA, USA) for 2 h at room temperature. Cells were washed to remove excess primary antibody, and incubated with the appropriate fluorescently labeled secondary antibodies for 1 h at room temperature. After mounting, fluorescence images were acquired using a confocal microscope.

### Western Blot Analysis

Cell lysates were collected and lysed in PRO-PREP (iNtRON BIOTECHNOLOGY, Korea) containing additional set of phosphatase inhibitors. Protein concentration was determined using a BCA assay kit. Proteins (30 μg/each conditions) were immediately heated for 5 min at 100°C. Each sample was subjected to SDS-PAGE on gel containing 12% (w/v) acrylamide under reducing conditions. Separated proteins were transferred to PVDF membranes (Millipore Corp., Billerica, MA, USA), and then the membranes were blocked with 5% skim milk. Membrates were developed using chemiluminescence assay kit (ECL; Millipore Corp., Billerica, MA, USA) and subsequently analyzed using Chemiluminescence Imaging System (Davinch-K, Seoul, Korea). Data were analyzed using Image J 1.38 software.

### Analysis of Mitochondrial Function and Mass

The mitochondrial membrane potential (ΔΨm) and mitochondrial mass of intact cells was measured as described previously [26]. Briefly, cells were washed with PBS and trypsinized. The protein concentration of cells was adjusted to 0.2 mg/mL in DMEM without phenol red (Life Technologies-Invitrogen), FBS and antibiotics. TMRE (200 nM) and MitoTracker Green FM (200 nM) was added to the cell suspension. Cells were incubated at 37°C for 30 min in the dark. ΔΨm and Mitochondria were measured by flow cytometry. TMRE fluorescence (582 nm) and MitoTracker Green FM (525 nm) were measured using the FL2 and FITC channel.

### Measurement of ROS Production

Level of intracellular hydrogen peroxide was determined by flow cytometry using 2′,7′-dichlorodihydroflurescein (H2DCFDA) (Invitrogen). Briefly, cells were treated with H2DCFDA for 30 min. At least 2×10^4^ cells were analyzed by a FACS Calibur flow cytometer (Becton-Dickinson, San Josè, CA, USA), with laser excitation set at 495 nm and a 525 nm emission filter to detect green fluorescence. The negative control was obtained omitting fluorescence probe-mix from the reaction and auto-fluorescence was estimated. To measure intracellular superoxide levels, the cells were incubated for 30 min in Krebs-HEPES buffer containing a dihydroethidium (DHE), washed twice, and analyzed using a FACS and confocal microscopy. Mitochondrial ROS levels were measured using the mitochondria specific fluorescent hydroethidine-derivative dye as previously described [27]. Cells were treated with glutamate with or without PFF and then incubated with MitoSOX for 30 min at 37°C in 5% CO2. The cells were washed twice with PBS, fixed with 4% paraformaldehyde, and analyzed by FACS and data were analyzed using the FlowJo.

### Transient Cerebral Ischemia Models and Intracerebral Ventricular Injection of PFF

Transient focal cerebral ischemia was induced by middle cerebral artery occlusion (MCAO) in rats. In brief, male rats were anesthetized with 1.5%to 2% isoflurane in a mixture of 30% O2:70% N2O for either a shorter duration with a face mask. The left external, internal, and common carotid arteries were exposed through a midline neck incision. After coagulating and cutting of the branches of the external carotid arteries, a 3–0 monofilament nylon suture with a blunted tip was inserted into the lumen of the external carotid artery, advanced into the internal carotid artery, and then into the origin of the middle cerebral artery. Reperfusion was conducted after 1 hour from inserting filament. An optimum dose of PFF (10 μg in 10 μl PBS) was infused by intracerebroventricular injection into the left lateral cerebral ventricle, once a day for 3 days before ischemia induction. Damage was assessed 24 h after MCAO by TTC staining. Damage was assessed 24 h after MCAO by TTC staining.

### Measurement of Infarction

All rats were sacrificed at 24 hours after ischemia under deep pentobarbital anesthesia, five coronal sections (2 mm thick) were stained with 2,3,5-triphenyltetrazolium chloride (TTC; Sigma, St. Louis, MO) to quantify infarct volumes. The degree of brain damage was estimated as the volumetric ratio of the ischemic side divided by the contra-lateral side using image j software (NIH, MD, USA).

### Cresyl Violet

Paraffin embedded tissues were cut as 5 μm. Sections were mounted onto poly-l-lysine coated slides and allowed to dry overnight. Slides were then dehydrated and defatted in 70, 95, and 100% ETOH, followed by rehydration and staining in the cresyl violet solution (Sigma, St Louis, MO, USA). Slides were rinsed in de-ionized H2O, again dehydrated, cleared with xylenes, and coverslips were applied using Mount G (Sigma-Aldrich, MO, USA). Sections were then visualized using a Leica SCN 400 (Leica Corp., Wetzlar, Germany) slide scanner.

### Statistical Analyses

All data were analyzed by Student’s *t*-test with Bonferroni adjustment or ANOVA for multiple comparisons and are presented as the means ± SD. Statistical comparisons were carried out using GraphPad Prism software (GraphPad Software, Inc. La Jolla, CA, USA) and the Tukey’s Multiple Comparison Test was used to determine one-way analysis of variance (ANOVA) procedures. Differences were considered significant at *p* <0.05.

## Results

### PFF Stimulation Has No Cytotoxic Effects in PC12 Cells

**Fig 1:**
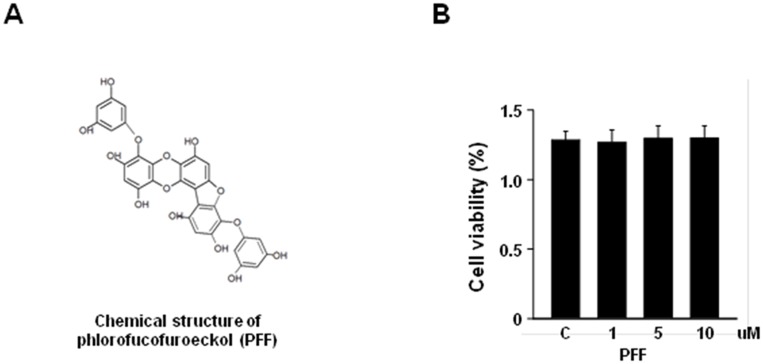
Cytotoxic effect of PFF in PC12 cells. (A) Chemical structure of PFF. (B) PC12 cells were treated with increasing concentrations of PFF or a vehicle control for 24 h. Cell viability was assessed with the MTT assay. Data represent the means and SD of three independent experiments. SC, vehicle control (0.01% DMSO).

We first isolated PFF from *Ecklonia cava* Kjellman (Fig 1A). Although the properties of PFF have been studied in neuronal cells [24, 25], the cytotoxic effect of *Ecklonia cava* Kjellman-derived PFF on PC12 cells has not been examined. We, thus, used the CCK8 assay to evaluate the dose-dependent cytotoxic effects of PFF on PC12 cells. We showed that stimulation with PFF had no significant effect on cell viability at various concentrations (1–10 μM) over 24 h (Fig 1B). Thus, we used PFF at a concentration of 10 μM in the following experiments.

### PFF Inhibits Glutamate-Induced Cell Death in PC12 Cells

**Fig 2:**
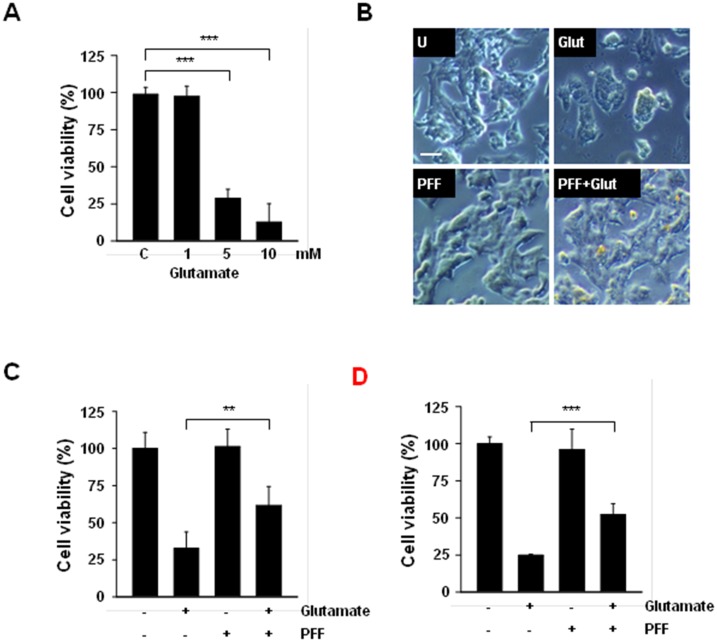
PFF treatment protected against glutamate-induced neurocytotoxic damage in PC12 cells. (A) PC12 cells were stimulated with glutamate (1, 5, 10 mM) or vehicle control for 24 h and cell viability then was measured with the MTT assay. (B and C) Undifferentiated PC12 cells were treated with PFF (10 μM) for 45 min before glutamate stimulation (5 mM). After 24 h, the neuroprotective effects of PFF were assessed with an inverted phase-contrast microscope (for morphological changes, B; scale bars = 25 μm) and the MTT assay (for cell viability, C). (D) Retinoic acid-mediated differentiated PC12 cells were incubated with PFF (10 μM) for 45 min before glutamate stimulation (5 mM). After 24 h, cell viability was analyzed by MTT assay. Data represent the means and SD of three independent experiments. *p < 0.05, ***p < 0.001 vs. the control group. SC, vehicle control (0.01% DMSO), Glut, glutamate.

Production of excessive extracellular glutamate and abnormal activation of is receptors are among the main causes of neuronal cell death during the progression of acute or chronic ischemic stroke. Thus, we investigated whether glutamate-induced neurotoxicity occurred in PC12 cells. Following glutamate stimulation, cell viability decreased markedly in a dose-dependent manner. We observed a reduction of 62% at 5 mM and one of 81% at 10 mM in glutamate-stimulated PC12 cells (Fig 2A). We next evaluated changes in cellular morphology and cell viability to examine the protective effects of PFF in glutamate-stimulated PC12 cells. As shown in Fig 2B, glutamate-stimulated PC12 cells exhibited a round, irregular morphology and a marked reduction in the number of neurons. However, PFF pretreatment effectively improved glutamate-mediated neuronal degeneration. Moreover, the glutamate-induced decrease in cell viability was also attenuated by pretreatment with PFF (Fig 2C). To explore whether glutamate-induced neuronal cell death was also attenuated by pre-treatment with PFF in differentiated, as well as undifferentiated, PC12 cells, PC12 cells were differentiated by treatment with retinoic acid (RA). As shown in Fig 2D, the exposure of differentiated PC12 cells to 5 mM glutamate was sufficient to cause neuronal toxicity; however, these effects were improved by pretreatment with PFF. These results indicate that PFF protects against glutamate-induced neurotoxicity in both undifferentiated and differentiated PC12 cells.

### PFF Exhibits Enhanced Neuronal Protection against Glutamate-Induced Cytotoxicity in a Caspase-Dependent Apoptosis Manner

**Fig 3:**
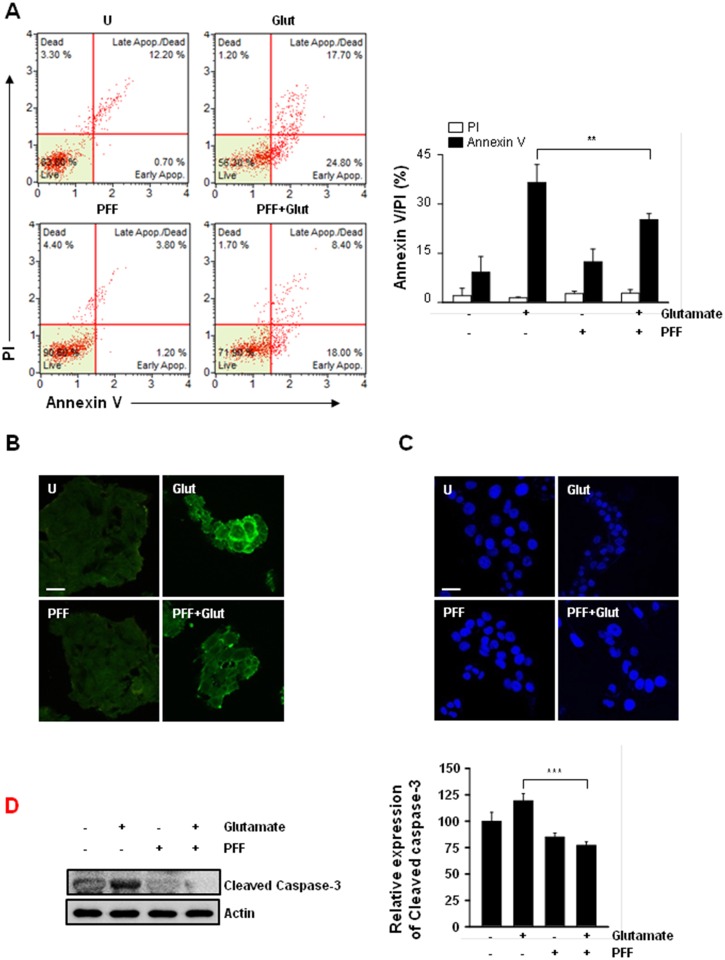
PFF enhanced survival in glutamate-stimulated PC12 cells via regulation of apoptotic cell death. PC12 cells were stimulated with glutamate (5 mM, 24 h) in the presence or absence of PFF (10 μM). (A–B) Cells were subjected to Annexin V/PI staining and analyzed by flow cytometry (A) and fluorescence microscopy (B; scale bars = 25 μm). (C) DNA fragmentation and nuclear condensation were detected by Hoechst 33258 staining under each condition. Scale bars = 25 μm. (D) Cell lysates were collected and then subjected to SDS-PAGE, followed by immunoblot analysis using anti-caspase-3 Abs. Actin was used as a loading control. SC, vehicle control (0.01% DMSO), Glut, glutamate.

Previous studies have found that glutamate exposure resulted in neuronal cell death through necrosis or apoptosis, depending on the neuronal cell type [28, 29]. To examine the inhibitory mechanisms of PFF in glutamate-induced cytotoxicity, PC12 cells were assessed by Annexin V and propidium iodide (PI) staining followed by flow cytometry (Fig 3A) or confocal microscopy (Fig 3B). Glutamate stimulation alone greatly facilitated the induction of apoptosis, including Annexin V^+^/PI^-^ (24.8%) and Annexin V^+^/PI^+^ populations (17.7%); however, AnnexinV^-^/PI^+^ populations were not altered. By comparison, pretreatment with PFF significantly inhibited glutamate-induced apoptosis (Annexin V^+^/PI^-^, 18.0%; Annexin V^+^/PI^+^ populations, 8.4%). Moreover, glutamate-induced DNA fragmentation was effectively attenuated by PFF pretreatment (Fig 3C). We further examined whether PFF regulated caspase-dependent cell death. As shown in Fig 3D, glutamate-induced cleavage of caspase-3, -8, and poly (ADP-ribose) polymerase (PARP) was inhibited in PC12 cells by pretreatment with PFF. These findings suggested that PFF treatment significantly improved neuronal degeneration through attenuation of caspase-dependent apoptosis in glutamate-stimulated PC12 cells.

### PFF Regulates Glutamate-Mediated Intracellular ROS Generation

**Fig 4:**
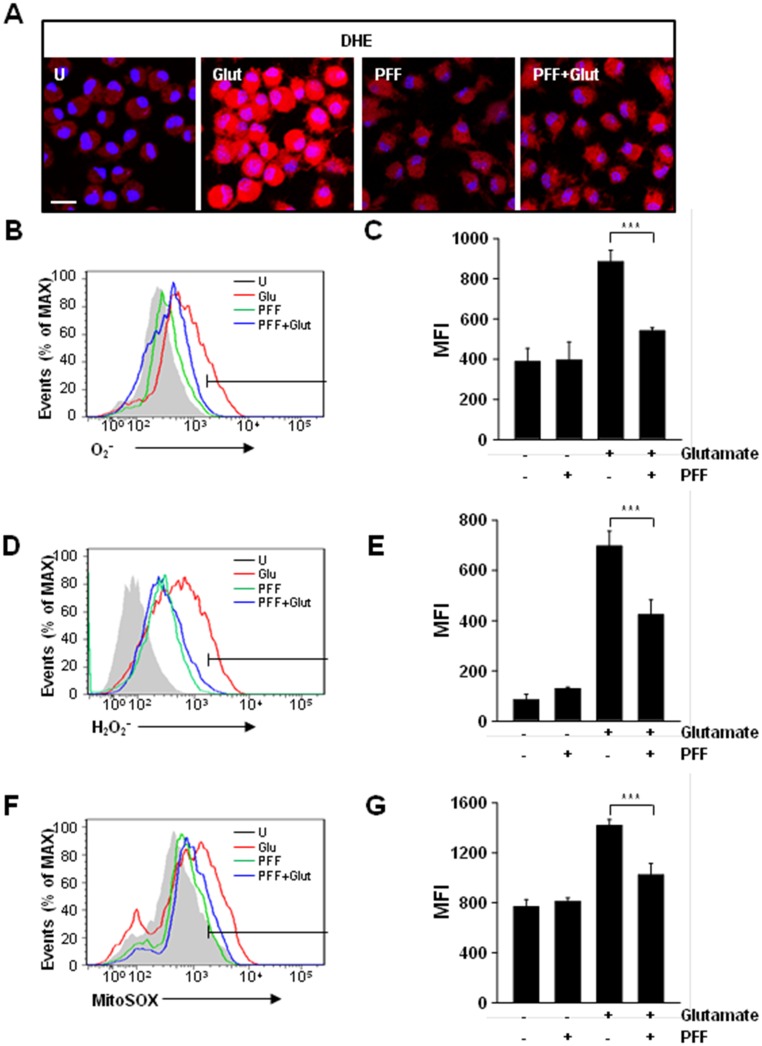
PFF regulated glutamate-induced ROS generation in PC12 cells. PC12 cells were pretreated with PFF (10 μM) for 45 min and then stimulated with glutamate (5 mM) for 24 h. (A–C) Cells were stained with DHE for 30 min to measure intracellular superoxide (O_2_^-^) using fluorescence microscopy (A; scale bars = 25 μm) and flow cytometry (B). (D and E) Cells were stained with H_2_DCFDA for 30 min to measure intracellular hydrogen peroxide (H_2_O_2_) using flow cytometry. (F and G) Cells were stained with MitoSOX for 30 min to measure mitochondrial ROS using flow cytometry. (C, E, G) The bar graph presents a quantitative analysis of the generation of intracellular superoxide (for C), H_2_O_2_ (for E), or mitochondrial ROS (for G). Data represent the means and SD of three independent experiments. ***p < 0.001 vs. the control group. SC, vehicle control (0.01% DMSO), Glut, glutamate.

Recently, several studies have reported that impaired generation of ROS, including superoxide (O_2_^•-^), hydroxyl radicals (HO^•^), and hydrogen peroxide (H_2_O_2_), was closely associated with caspase-dependent and/or -independent neuronal cell death [30, 31]. Thus, we determined whether PFF was involved in the negative regulation of oxidative stress. PC12 cells were stimulated with glutamate in the presence or absence of PFF, and ROS generation was determined using dihydroethidium (DHE; Fig 4A–4C) or 2,7-dichlorodihydrofluorescein-diacetate (DCFH-DA; Fig 4D and 4E) as a probe for confocal microscopy and flow cytometric analyses. Glutamate alone caused the strong intracellular generation of O_2_ and H_2_O_2_, whereas glutamate-induced generation of ROS in PC12 cells was attenuated significantly by PFF pretreatment.

We further characterized the inhibitory role of PFF in glutamate-mediated mitochondrial ROS generation using the mitochondrial-specific superoxide indicator MitoSOX, which is oxidized by superoxide and then induces an increase in red fluorescence. Mitochondrial ROS increased significantly after treatment with glutamate (Fig 4F and 4G), however, glutamate-induced Mitochondrial ROS generation also was decreased by pretreatment with PFF. These results suggested that PFF protected against glutamate-mediated neuronal cell death by regulating the generation of cytosolic and mitochondrial ROS.

### PFF Improves Glutamate-Induced Mitochondrial Dysfunction

**Fig 5:**
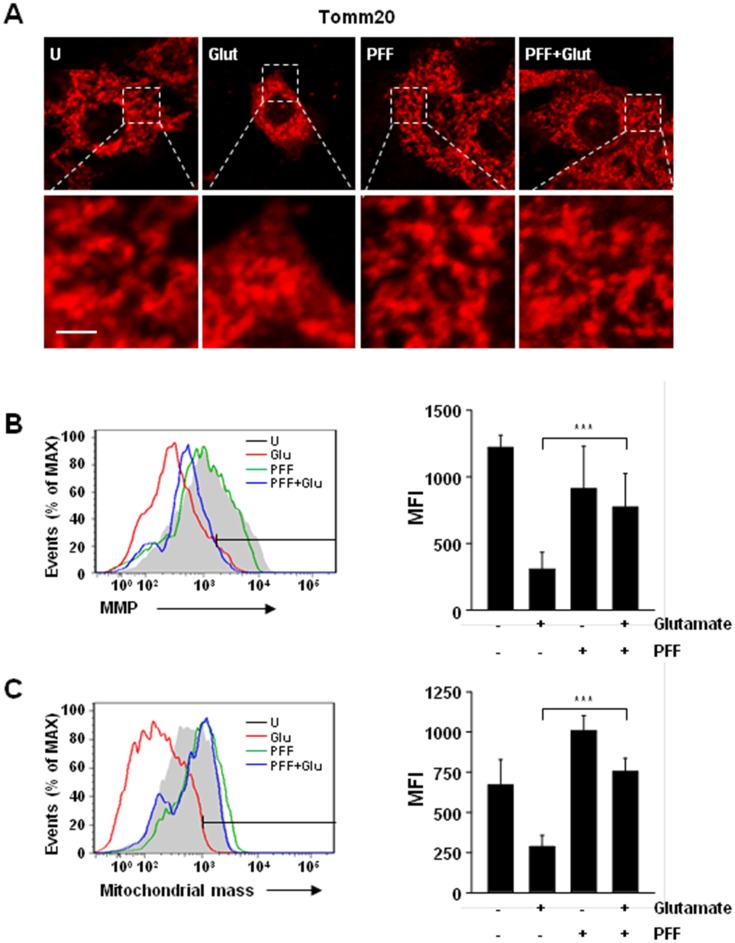
Glutamate-induced mitochondrial dysfunction in PC12 cells was improved by PFF treatment. PC12 cells were stimulated with glutamate (5 mM, 24 h) in the presence or absence of PFF (10 μM). (A) Cells were immunolabelled with a Tomm20 antibody, followed by the addition of Cy3-conjugated secondary antibody. Representative immunofluorescence images (scale bars = 10 μm). (B) Mitochondrial membrane potential (ΔΨm) was measured under the indicated conditions using the ΔΨm-sensitive fluorochrome MitoTracker Red CMXRos and flow cytometric analysis. (C) MitoTracker fluorescence signals for mitochondrial mass were measured using flow cytometric analysis. (Left in B and C) Representative histogram from three independent replicates. (Right in B and C) Bar graph shows ΔΨm (B) or mitochondrial mass (C) mean fluorescence intensities. Data represent the means and SD of three independent experiments. ***p < 0.001 vs. the control group. U, untreated condition. Glut, glutamate.

Accumulation of impaired mitochondria is one of the major causes not only of the induction of the intrinsic apoptotic pathway but also of the generation of mitochondrial ROS [32–34]. To investigate whether the glutamate-mediated increase of abnormal mitochondria was rescued by PFF treatment, we first evaluated mitochondrial morphology using Tomm20 (mitochondrial outer membrane) staining (Fig 5A). Mitochondrial morphology was altered at 12 h and sustained at 24 h in glutamate-stimulated PC12 cells. However, the increased mitochondrial damage was attenuated significantly by PFF treatment (Fig 5A).

We next evaluated mitochondrial membrane potential (ΔΨm) using flow cytometry. The glutamate-stimulated PC12 cells showed decreased ΔΨm; however, the decrease was significantly reduced in PFF-pretreated cells, as well as in unstimulated cells (Fig 5B). In addition, mitochondrial mass was decreased, by about 25%, in glutamate-stimulated PC12 cells, but this was rescued by PFF pretreatment (Fig 5C). These findings suggested that the PFF improved glutamate-mediated mitochondrial dysfunction, which was mediated by rescue of ΔΨm and mitochondrial mass.

### PFF Protects against Ischemic Brain Injury in Rats

**Fig 6:**
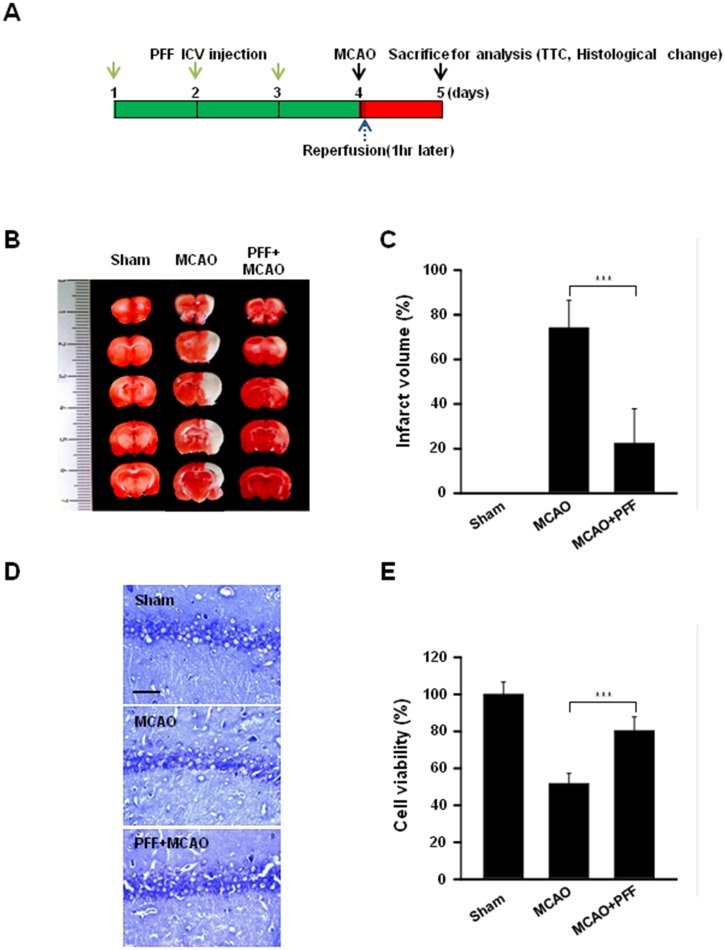
PFF treatment prevented MCAO-induced brain damage in a rat animal model of ischemic stroke. (A) *In vivo* experimental schedule. PFF was injected intracerebroventricularly into the left lateral cerebral ventricle of the rat, as described in the Materials and Methods section. (B and C) TTC staining was used to assess infarction volume in coronal sections. (B) Representative images of coronal sections from rats receiving sham, MCAO, and PFF plus MCAO on day 5. The red region indicates intact tissue, and the white region indicates the infarct area. (C) The bar graph represents the means ± SD of infarct volume under each condition. (D and E) Histological changes in the hippocampus were observed using cresyl violet staining. (D) Representative images of each section. Scale bars = 25 μm. (E) Cell viability was determined in tissue sections from each rat group. ***p < 0.001 vs. the control group.

Although there are several experimental *in vivo* stroke models in various species, including rodents, cats, canines, and even primates, the middle cerebral artery occlusion (MCAO) model is well known as a usable experimental focal cerebral ischemia model in rodents [35]. To evaluate the *in vivo* protective effects of PFF, PFF was administered intracerebroventricularly (ICV) three times for 3 consecutive days before ischemia/reperfusion, as described in Fig 6A. When compared with those of the sham group, the coronal infarct volumes of the rats receiving ischemia/reperfusion were greatly increased; however, these were significantly decreased, by about 72% (p < 0.001), in the PFF-administered rats (Fig 6B and 6C). Moreover, we used histological staining, with hematoxylin and eosin (H&E), to evaluate the cellular responses against focal ischemic brain injury associated with PFF administration (Fig 6D and 6E). The brain tissues suffering ischemia/reperfusion showed severe neuronal damage, such as neuronal shrinkage, apoptotic tissue injury, and increased nuclear basophilia; however, these phenotypes were effectively diminished in rats administered PFF. These results indicated that local ICV injection of PFF significantly reduced infarct volume and severe cellular responses *in vivo* in a MCAO model.

## Discussion

Ischemia/reperfusion has been reported to be closely associated with various disorders, including stroke, myocardial infarction, hypovolemic shock, and peripheral vascular disease [36, 37]. Following thrombolytic therapy for ischemic stroke, ischemia/reperfusion-mediated neurological damage occurs as a result of insults to cerebral microcirculation, such as oxidative bursts, activation of inflammatory mediators, capillary hypoperfusion, and apoptosis of neuronal cells [38]. Although the direct association between an increased release of glutamate and subsequent cell death during ischemia has not been elucidated fully, it is an important cause of neuronal injury [39]. Additionally, we showed that PFF effectively contributed to neuronal protection against glutamate stimulation in both undifferentiated and differentiated PC12 cells (Fig 2C and 2D). Furthermore, we evaluated the *in vivo* protective effect of PFF following ischemic injury caused by MCAO occlusion and sham procedures (Fig 6A). The results showed that intracerebroventricular injection of PFF significantly improved MCAO-induced brain injury *in vivo* (Fig 6B and 6C), and this effect was related to the inhibition of neural cell death (Fig 6D and 6E). These data suggest that PFF administration results in inhibition of stroke-associated brain damage and increased cell viability.

Reactive oxygen species (ROS) are involved in various physiological and pathological events, including aging, cancer, inflammation, host immune responses, and neurodegeneration [12, 14, 34, 40]. Recent studies demonstrated that the moderate generation of intracellular ROS acts as an essential intermediate for the modulation of diverse signal transduction pathways [41]. However, excessive generation of ROS by uncontrolled oxidative stress resulted in a wide spectrum of neurological disorders via the oxidative modification of essential components; this can cause not only neuronal cell death but also the organism’s death [42]. Thus, there have been various trials on the possible use of anti-oxidative agents as neuroprotective drugs against ischemic stroke. For example, administration of gramicidin A, a glutamate-substituted analog, led to an amelioration of neural cell death and brain injury following ischemia/reperfusion by maintenance of the mitochondrial membrane potential [43]. Additionally, NaHS, a source of endogenous gaseous mediator, showed potent protective effects in an experimental rat model of cerebral ischemia/reperfusion injury. The protective effect was mainly mediated by anti-oxidative effects, including increased SOD activity and decreased mRNA expression of p47phox and gp91phox subunits of NADPH oxidase [44]. We also found that PFF significantly suppressed glutamate-induced generation of both intracellular superoxide (Fig 4A–4C) and hydrogen peroxide (Fig 4D and 4E). Previous studies showed that solvent fractions isolated from the edible brown alga *Ecklonia bicyclis* that contained dieckol and PFF-A exhibited anti-oxidative activity and demonstrated their possible use as candidate drugs for the treatment of oxidative stress-associated disorders, such as Alzheimer’s disease [25, 45]. Moreover, several studies have supported the beneficial role of polyphenols as antioxidants by identifying their various mechanisms of action, including scavenging of free radicals and inhibition of intracellular ROS generation, thereby preventing the development of cancer, neurodegenerative disorders, diabetes, and inflammatory diseases [46–48]. Our results extend these findings and suggest that PFF may be a candidate preventative drug for ischemic stroke via regulation of excessive ROS generation. Taken together, the current data provide insights into the role of ROS in neuronal cell death in ischemic stroke and into the potential therapeutic uses of PFF.

Considerable evidence suggests that mitochondria are an essential organelle involved in diverse physiological pathways, including energy metabolism, immune regulation, and cellular homeostasis [49–51]. Mitochondria are also major sources of superoxide and hydrogen peroxide through various enzymes, including complex I (NADH dehydrogenase), complex III (coenzyme Q and cytochrome *c* oxidoreductase), and manganese superoxide dismutase (MnSOD) [34, 52]. In addition, mitochondria-derived ROS are also produced by alternative mitochondrial pathways, such as growth factor adaptor Shc [53], nicotinamide adenine dinucleotide phosphate oxidase 4 (Nox4) [54], monoamine oxidase (MAO), and α-ketoglutarate dehydrogenase [51]. Although the role of the mitochondrial ROS generated by these enzymes in diverse physiological and/or pathological situations is controversial, recent studies have shown that overproduction of mitochondrial ROS during ischemia and reperfusion plays a key role in pathogenesis and progression [8, 10, 52]. We found that glutamate-mediated mitochondrial ROS generation was increased in PC12 cells; however, these effects were significantly attenuated by pretreatment with PFF. Importantly, mitochondrial dysfunction is closely associated with the pathogenesis of various neurodegenerative diseases, including stroke, Alzheimer’s, Parkinson’s, and Huntington’s diseases [2, 12]. Previous studies have demonstrated that mitochondrial morphology is fundamentally regulated by exquisite maintenance of fusion and fission processing; however, overproduction of glutamate or amyloid-β resulted in destruction of this balance through changes in the levels of mitochondrial fusion- and/or fission-associated proteins, such as MFN2 and DLP1 [55–57]. In this study, we showed that PFF treatment improved glutamate-induced mitochondrial dysfunction in PC12 cells. Confocal analysis of PC12 cells using translocase of the outer mitochondrial membrane 20 (‘Tomm20’), which is found mainly on the outer mitochondrial membrane, indicated that glutamate-induced mitochondrial fragmentation and morphological changes were inhibited in PFF-pretreated cells (Fig 5A). Consistent with the confocal microscopic images, flow cytometry analysis showed that the glutamate-induced decrease in mitochondrial membrane potential and mass also recovered with PFF pretreatment (Fig 5B and 5C). Thus, the neuroprotective effect of PFF is mediated by improvement in mitochondrial function.

**Fig 7:**
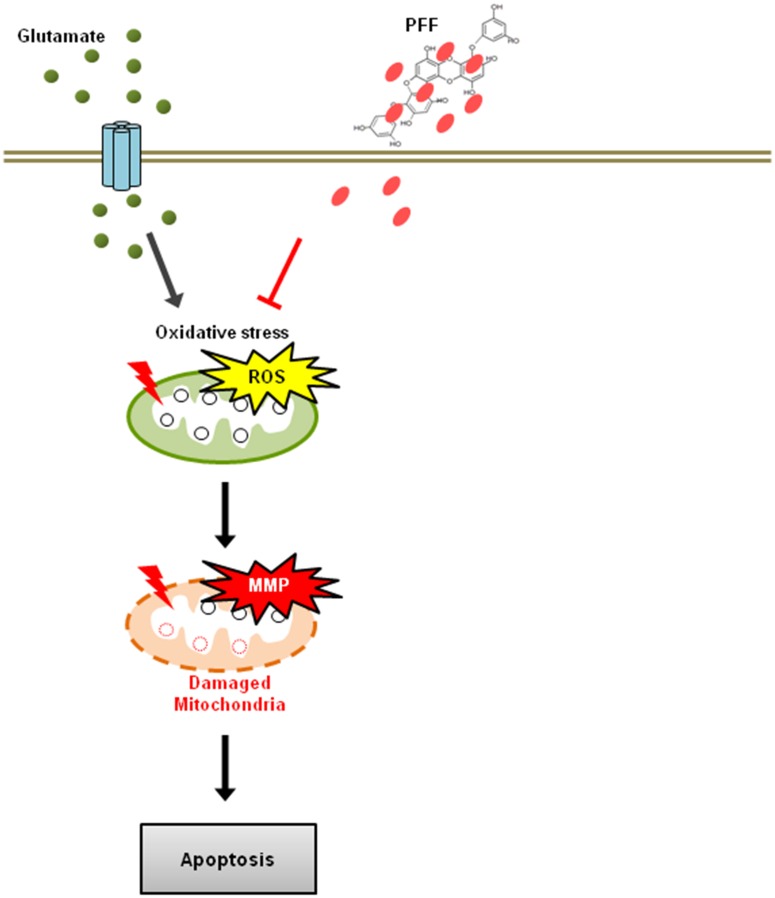
A schematic model depicting the neuroprotective roles of PFF in an ischemic stroke model. Glutamate caused disturbances in the oxidant/antioxidant balance in the cellular system and then resulted in a permeability transition of the mitochondrial membrane. This further altered the translocation of the mitochondrial death-signaling pro/anti-apoptotic proteins, such as Bax, Bcl-2, and cytochrome *c*, resulting in the activation of the caspase cascade and DNA damage, culminating in cell death. Administration of PFF helped to maintain the oxidant/antioxidant balance and alter the changes in the mitochondria-mediated apoptotic signaling cascade that resulted in neuronal cell damage.

Bioactive derivatives isolated from *Ecklonia* species have shown multifunctional properties, including anti-cancer, anti-inflammatory, and anti-diabetic effects [15]. For example, dieckol isolated from *Ecklonia cava*, one of the phlorotannin polyphenol compounds, regulated oxidative stress and apoptosis to protect against the glucotoxicity of hyperglycemia [58]. Dieckol also resulted in suppression of ovarian tumor cells through the activation of caspase-dependent apoptosis [59]. Additionally, PFF-A isolated from *Ecklonia stolonifera* exhibited cytoprotective effects through inhibition of caspase-dependent apoptosis in tacrine-treated HepG2 cells [60]. Our results indicated that glutamate-stimulated PC12 cells showed increased apoptosis, DNA fragmentation, and caspase activation. However, PFF significantly inhibited glutamate-induced upregulation of these effects (Fig 3). These data suggest that PFF increases neuronal cell survival through inhibition of both early and late apoptosis, as well as regulation of ROS generation. Our proposed model for the protective pathway of PFF is summarized in Fig 7.

In conclusion, the results of current study underscore the potential of PFF as a neuroprotective agent in ischemic stroke and showed that localized treatment with PFF effectively suppressed brain injury in this condition. These findings suggest that adding a supplement from *Ecklonia* species and from other candidates containing PFF to clinical drugs may be useful for protecting against or treating various neurodegenerative diseases.
